# Effects of transcranial direct current stimulation on grip force control in patients with cerebellar degeneration

**DOI:** 10.1186/s40673-017-0072-8

**Published:** 2017-09-15

**Authors:** Liane John, Michael Küper, Thomas Hulst, Dagmar Timmann, Joachim Hermsdörfer

**Affiliations:** 1 Department of Neurology, University Hospital Essen, University of Duisburg-Essen, Essen, Germany; 2 Erasmus University College, Rotterdam, The Netherlands; 3 Department of Sport and Health Sciences, Institute of Movement Science, Technical University of Munich, Munich, Germany

**Keywords:** After effects, Motorcortex, Cerebellum, Direct current

## Abstract

**Background:**

The control of grip forces when moving a hand held object is impaired in patients with cerebellar degeneration. We asked the question whether after-effects of anodal transcranial direct current stimulation (tDCS) applied to the lateral cerebellum or M1 improved grip force control in cerebellar patients.

**Methods:**

Grip force control while holding an object during cyclic arm movements was assessed in patients with pure cerebellar degeneration (*n* = 14, mean age 50.2 years ± SD 8.8 years) and age- and sex-matched control participants (n = 14, mean age 50.7 years ± SD 9.8 years). All subjects were tested before and after application of tDCS (2 mA, 22 min) in a within-subject design. Each subject received anodal tDCS applied to the cerebellum, anodal tDCS applied to M1 or sham-stimulation with a break of 1 week between the three experimental sessions.

**Results:**

There were no clear after-effects of tDCS on grip force control neither in control participants nor in cerebellar patients. Cerebellar patients showed typical impairments with higher grip forces, a higher variability of movements.

**Conclusion:**

In the present study, deficits in grip force control were neither improved by tDCS applied over the cerebellum nor M1 in cerebellar degeneration.

## Background

While moving hand-held objects, inertial loads arise from the acceleration of the object mass and grip forces must prevent slippage of the object despite the destabilizing loads [1]. In healthy subjects, grip and load force change in parallel indicating that the grip force is adjusted in a predictive way in order to compensate changing load forces [2, 3]. Apart from load perturbations grip force control depends on physical object properties such as weight, shape and surface friction [4, 5]. During free movement of a hand held object cerebellar patients typically show slower movements, higher peak grip forces and higher movement variability compared to healthy controls [1, 6]. Less efficient coupling of grip and load forces was reported in some studies [7, 8] but was not detected in others [1, 6]. The impaired grip force control found in cerebellar patients likely adds to patients’ disability in everyday-life. As yet, controlled studies are lacking of rehabilitative interventions to improve grip force control in cerebellar patients.

Because of its ability to modify cerebellar excitability and to induce plastic modifications without significant side effects, cerebellar transcranial direct current stimulation (tDCS) may be a powerful tool in the rehabilitation of cerebellar patients [9]. As yet, it is unknown whether tDCS improves deficits of cerebellar patients in grip-force control.

A seminal study conducted by Galea et al. [10] demonstrated that anodal cerebellar tDCS led to faster visuomotor-adaptation in young and healthy subjects. Galea et al. applied anodal stimulation over the right cerebellum during the experimental task (“online stimulation”) [2]. In a recent sham-controlled study, a single session of cerebellar anodal tDCS was followed by significant improvement of ataxia [11] as assessed by the Scale for the Assessment and Rating of Ataxia (SARA; [12]) and the International Cooperative Ataxia Rating Scale (ICARS; [13]). Importantly, there was also a better performance in the 9-hole peg test as a marker of upper limb coordination and finger dexterity. These results were replicated in a study looking at long-term effects. Anodal tDCS was applied 5 days a week for 2 weeks. Positive effects were still present after 3 months [14]. In both studies anodal stimulation was applied over the cerebellum bilaterally. Outcome measures were assessed after the stimulation (“offline stimulation”).

TDCS of the motor cortex may also be helpful in treatment of cerebellar disease because motor cortex excitability is reduced in patients with cerebellar disease [15, 16]. Bilateral M1-stimulation was followed by improvement of ataxic gait with better symmetry of step execution and reduction of base-width in three patients with cerebellar disease [17] (anodal electrode placed over M1 contralateral to the most affected side, offline stimulation). Authors also reported improvements in the SARA score for upper limb function. Therefore, M1 stimulation maybe another promising approach to improve grip-force modulation in cerebellar patients.

Few studies have assessed the effects of tDCS on the learning and retention of the control of grip forces. One study found a better reproduction of an irregular force pattern in participants who received 5 days of anodal tDCS over the contralateral M1 (offline stimulation). Consolidation of performance was improved over the night in the tDCS group [18]. Other studies reported heterogeneous findings, such as absent effects of anodal tDCS stimulation over contralateral M1 (online and offline stimulation) in a grip force tracking task [19] or performance decrements in the form of increased variability in an isometric constant grip force task [20]. Investigating the grasping and lifting of objects with different surface material in elderly subjects, the authors reported a decrease of the grip force in those subject who practiced a different fine motor task during tDCS stimulation of contralateral M1 compared to subjects who practiced with sham stimulation [21]. The effect of stimulation was particularly evident with a more slippery surface and less with the rougher surface and the timing of the lifting act was not affected. Interestingly, the application of dual hemisphere tDCS (online and offline stimulation) in a group of stroke patients resulted in a decrease of grip forces and a decreased time to establish the grip during a similar grasping and lifting task [22]. Heterogeneous effects of tDCS over M1 have been reported in healthy subjects, elderly subjects or stroke patients. As yet, effects of tDCS on disordered grip force control in cerebellar patients are lacking.

In the present study we assessed the effect of tDCS applied to the cerebellum or M1 on grip-force modulation during self-generated, sinusoidal up-down movements with a handheld object in healthy subjects and patients with cerebellar degeneration. We hypothesized that anodal tDCS over the cerebellum or M1 improves grip-force control in cerebellar patients. We expected higher movement frequencies, lower grip forces and more precise coupling after stimulation in the patient group.

## Methods

### Participants

Fourteen individuals with pure cerebellar degeneration (6 females; mean age ± SD of 51.2 ± 7.6 years) and 14 age-matched controls without any known neurological diseases (5 females; mean age ± SD 50.8 ± 10.1 years) participated in this study. All subjects were right-handed as assessed by the Edinburgh Handedness Inventory [23]. A summary of the subjects’ characteristics can be found in Table 1. The severity of cerebellar symptoms in cerebellar participants were assessed by two experienced neurologists (DT & MK) based on the International Cooperative Ataxia Rating Scale (ICARS; (13)) and the Scale for the Assessment and Rating of Ataxia (SARA; (12)). Five cerebellar participants had a genetically defined spinocerebellar ataxia (SCA6, SCA14). Five participants presented with autosomal dominant cerebellar ataxia (ADCA) type III. Three cerebellar participants had sporadic adult onset ataxia of unknown etiology (SAOA). One cerebellar participant presented with cerebellar degeneration caused by cerebellitis. These disorders are known to primarily affect the cerebellum [24, 25]. All subjects gave informed oral and written consent. The experiment was approved by the ethics committee of the medical faculty of the University of Duisburg-Essen and conducted in accordance with the Declaration of Helsinki. This study was conducted as part of another study investigating the direct tDCS effects on reach adaptation [26].

**Table 1:** Overview Cerebellar subjects and Control subjects

Cerebellar subjects							Controls		
ID	Age	Sex	Diagnosis	Disease duration	ICARS (total/100)	ICARS UL (total/20)	ID	Age	Sex
P01	30	M	SAOA	9 years	38.5	7.5	C01	28	M
P02	47	M	ADCA III	12 years	43.5	4.5	C02	33	M
P03	47	M	ADCA III	17 years	32.5	4.5	C03	47	M
P04	48	F	ADCA III	28 years	19	1	C04	47	M
P05	48	M	SCA 14	25 years	20	3	C05	50	F
P06	50	F	SCA 14	17 years	17	1	C06	51	F
P07	52	M	ADCA III	6 years	19.5	3	C07	52	M
P08	53	M	Cerebellitis	10 years	46	5	C08	54	M
P09	54	F	SCA 14	25 years	27	3.5	C09	55	M
P10	54	F	SAOA	18 years	31	4.5	C10	55	M
P11	55	M	SAOA	18 years	48	5	C11	55	F
P12	58	F	SCA 6	8 years	43.5	10	C12	57	F
P13	60	F	ADCA III	13 years	23	11	C13	63	F
P14	61	M	SCA 6	4 years	9	0	C14	65	M

Cerebellar subjects were age-matched with the control subject on the right side of the table. SCA6 = spinocerebellar ataxia type 6; SCA14 = spinocerebellar ataxia type 14; SAOA = sporadic adult onset ataxia; ADCA III = autosomal dominant ataxia type III; ICARS = International Cooperative Ataxia Rating Scale [13]. ICARS UL = score of right upper limb in finger-to-nose test, finger-to-finger test, pronation/supination and Archimedes spiral drawing. Disease duration is years since presentation of the first symptoms

### Task

All subjects participated in a task designed to analyze grip force adjustments according to movement induced load changes while holding an object. The task has been introduced by Flanagan and Wing [2]. The set-up in the present study has been used by Brandauer et al. in previous studies [1, 2].

Subjects’ grasped a custom-made instrumented object with their right hand. The object had a rectangular form with two grasping surfaces (60 × 60 mm) and a width of 26 mm. The grasping surfaces were covered with medium grain sandpaper (No. 240).

The object incorporated sensors to record the grip force on each side (0–100 N, accuracy ±0.1 N), the linear vertical and horizontal accelerations tangential to the graspingsurfaces (±50 m/s^2^, accuracy ±0.2 m/s^2^), and the load force (0–60 N,accuracy ±0.1 N).

The grip force of both sensors for each side was averaged. To increase the amplitude of the movement-induced sinusoidal load changes a weight of 300 g was fixed to the object which increased the total weight of the object to 500 g. Vertical acceleration (AccZ) was defined as pure kinematic acceleration due to movement. The net load force was calculated as the vectorial sum of weight (m x g), acting vertically, and the acceleration-dependent inertial loads in the vertical and sagittal directions (m xAccZ, m xAccY), acting tangential to the grip surfaces {LF = m x[(AccZ + g)^2^ + AccY^2^]^1/2^}.

Participants were asked to grasp the object and to hold it with the right hand in front of their trunk with grip surfaces vertical and parallel to their front. This orientation was kept constant during the movement. It was required to grasp the center of the object with the thumb on one side and the index and middle fingers on the opposite side. The three-finger grip was used to minimize rotational torques that arise when the object is grasped away from the center of mass.

After a verbal command subjects had to move the object along a vertical line up and down with an amplitude of about 30 cm at a frequency of about 0.8 Hz, which was demonstrated by the examiner sitting opposite to the subject by moving the hand up and down. The accurate movement execution was visually monitored by the examiner.

Following one practice trial, five trials of 22 s duration were performed successively.

### Data analysis

As the first step of data analysis, the first 2 s of each trial were discarded and the remaining 20 s divided into two 10 s-intervals so that 10 intervals per condition resulted.

The following measures were determined for each intervals:

1. Movement frequency and vertical acceleration
2. Variation of maximal/minimal acceleration during up/down movements as a measure for arm movement variability
3. Peak grip force levels
4. Coupling of grip and load forces

To quantify the performance in each interval, a computer algorithm first searched for peaks (local maxima and minima) in the sinusoidal profile of the vertical acceleration. Positive acceleration peaks corresponding to load force peaks occur at the lower turning point of the movement, negative accelerations and minimum loads occur at the upper turning point. The magnitude of vertical acceleration was calculated as the averaged acceleration range between positive and negative acceleration peaks. Variability was calculated as the standard deviation of positive and negative acceleration peaks within each interval (averaged for positive and negative peaks) related to the vertical acceleration magnitude described above. Movement frequency was determined from the power spectra of the acceleration profile.

To quantify the magnitude of the produced grip forces, the grip force peaks were determined ina window around each load force peak. In addition, minima of the ratio between grip-force and load-force were determined in the windows. Both values were averaged for each 10 s-interval. The force ratio represents a measure of the efficiency of the grip-force output related to the load.

The coupling between the modulation of grip-force and load-force was evaluated by calculating the cross-correlation function between both time series. The maximum cross-correlation coefficient was taken as the indicator of the precision of the coupling.

The resulting data values were averaged across the intervals of each participant and each condition.

### tDCS

Participants were invited for three experimental sessions separated by 1 week. In two sessions, subjects received verum tDCS stimulation, in one session sham stimulation. Anodal tDCS was performed over M1 and over the cerebellum. Sham tDCS stimulation was performed either over M1 or cerebellum. The order of the three sessions was counterbalanced between participants.

The grasping task was performed as part of another study [26]. In that study tDCS was applied during reach adaptation. The grasping task was performed before the reach adaptation task (and therefore prior tDCS) and after the reach adaptation task. The second testing took place on average 10:52 min (mean, ± 1:34 min SD) after the end of tDCS in patients, and 9:52 min (mean, ± 3:55 min SD) in the control group.

Stimulation parameters were chosen in close accordance with previous studies of Galea et al. [10, 27]. Anodal tDCS was delivered through two rubber electrodes (5 cm × 5 cm; surface area: 25 cm^2^) covered with conductive paste (Ten20 Conductive; Weaver) via a NeuroConn device (DC-Stimulator PLUS; NeuroConn). For cerebellar stimulation the anodal electrode was placed over the right cerebellar cortex, with the center of the electrode being 3 cm lateral to the inion, and the cathodal electrode was placed on the right buccinator muscle. The anodal electrode for M1 stimulation was centered over the area of the left primary motor cortex which elicited a response of the first dorsal interosseous muscle after single transcranial magnetic stimulation (TMS) pulses. TMS was delivered by a MagPro magnetic stimulator (MagPro; Dantec). The cathodal electrode was placed on the skin overlying the contralateral supraorbital region. During each experimental session, the electrodes were placed over all four stimulation locations, so participants were blinded for stimulation location.

In both cerebellar and M1 anodal stimulation, the target stimulation intensity was set at 2 mA, resulting in a current density of 0.08 mA/cm^2^. Current was ramped up from 0 mA to 2 mA in a period of 30 s. At the end of tDCS stimulation, current was ramped down from 2 mA to 0 mA in 30 s. In sham stimulation current was ramped-up in 30 s, remained at 2 mA for a duration of 60 s, after which current was ramped down again.

On average, subjects were stimulated for 25:34 min (mean, ± 6:34 min SD) in the patient group and for 21:37 min (mean, ± 2:32 min SD) in the control group**.**

One experimenter (LJ) ran all the behavioral experiments and used a prepared set of stimulation codes in order to remain blinded for stimulation polarity (sham or anodal). An experimenter (BB) who was not involved in the collection of behavioral data, deblinded the stimulation codes after data collection had ended.

### Statistical analysis

To assess the differences between the single-task conditions, repeated-measures ANOVAs were calculated with the between-subjectfactor “group” (controls, patients) and the within-subject factors “stimulation” (cerebellum, M1, sham) and “time” (pre stimulation, post stimulation). We expected to find differences between patients and control subjects obvious as effects (main and interactions) involving the factor “group” for the different measures. In addition, we expected that the ANOVA reveals effects of tDCS stimulation obvious as interactions between “stimulation” and “time” and also as a three way interaction to indicate differences in the effects of stimulation between patients and control subjects. T-tests were used for post hoc analyses**.** An alpha level of 0.05 was chosen to indicate statistical significance.

Intervals were excluded from statistical analyses if movements were performed very slowly (movement frequency < 0.3 Hz, 0.4% of 10 s-intervals) or if values of behavioral measures were out of two standard deviations of the mean (14,3% 10 s-intervals in controls excluded, 12,9% 10 s-intervals in patients excluded). The number of excluded intervals in each subject and condition never exceeded three, resulting in a minimum of seven data values that were averaged for each condition. In one patient, pre-stimulation data for the M1 session were missing preventing the inclusion of the subject in the statistical analysis. Acceleration data were missing due to technical problems in another patient for sham stimulation (pre and post tDCS).

## Results

### Performance of single patient

**Fig. 1:**
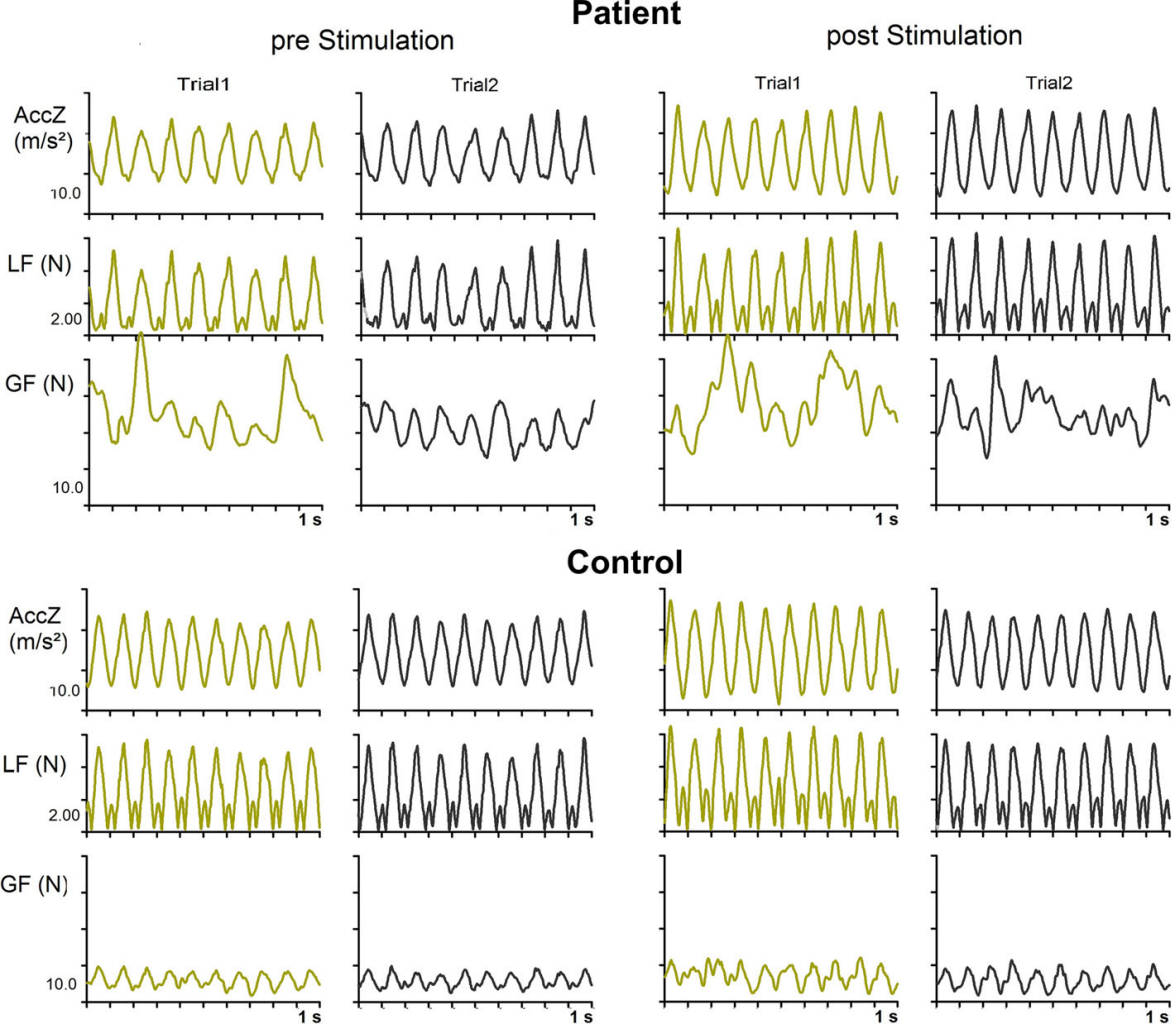
Vertical acceleration (AccZ), load force (LF) and grip force (GF) during 10 s intervals of continuous cyclic up-and-down movements of the grasped manipulandum. The first interval (2–12 s) of two out of five test trials before and after cerebellar tDCS of one individual patient and one healthy control subject is shown

Figure 1 shows the profiles of the vertical acceleration of the grasped object (AccZ), the combined gravitational and inertial load that result from the movements (LF) and the produced grip force (GF) in one patient and in one healthy control subject before and after the anodal stimulation of the cerebellum. The patient moved faster after the stimulation as obvious from higher accelerations. The patient’s grip force profile is clearly more irregular than the load force profile indicating decreased precision of the coupling between both forces. Nevertheless, most grip force peaks coincide in time roughly with main peaks of the load force profiles indicated some preservation of anticipatory control of the grip force. In the control subject, the grip force profile is regular and the timing of grip force peaks anticipates the loads force peaks. The magnitudes of the grip force peaks are substantially higher in the patient compared to the control subject. In general, the individual patient’s behavior reflects the performance of the patients’ group. No clear changes of grip force control were obvious before and after the stimulations and for the different stimulation conditions (see below).

### Group data

Subjects produced cyclic movements with frequencies slightly lower than instructed (overall data including patients and controls prior and post stimulation: mean 0.73 ± 0.17 Hz). Movement frequencies were somewhat higher for controls compared to patients without statistical significance (patients: 0.69 ± 0.16 Hz, controls: 0.76 ± 0.15 Hz, main effect of “group”: *P* > 0.1). The only significant effect was an increase of frequency after the stimulation compared to pre-stimulation values for all participants including patients and controls (pre tDCS: 0.72 ± 0.16 Hz, post tDCS: 0.74 ± 0.15 Hz, main effect of “time”: *F(1,25)* = 7.8, *P* = 0.010). The ANOVA results for the magnitude of arm acceleration reflected the findings for the frequency with higher accelerations produced post-stimulation (pre-tDCS: 11.3 ± 4.6 m/s^2^, post-tDCS: 13.0 ± 4.7 m/s^2^, main effect of “time”: *F(1,24)* = 42.9, *P* < 0.001) regardless from the group and whether cerebellar, M1 or sham stimulation was applied (all other main effects and interactions: *P* > 0.1). Thus, the kinematics of arm movements and consequently also the self-generated loads where comparable in magnitude between patients and control subjects as intended by the procedure.

**Fig. 2:**
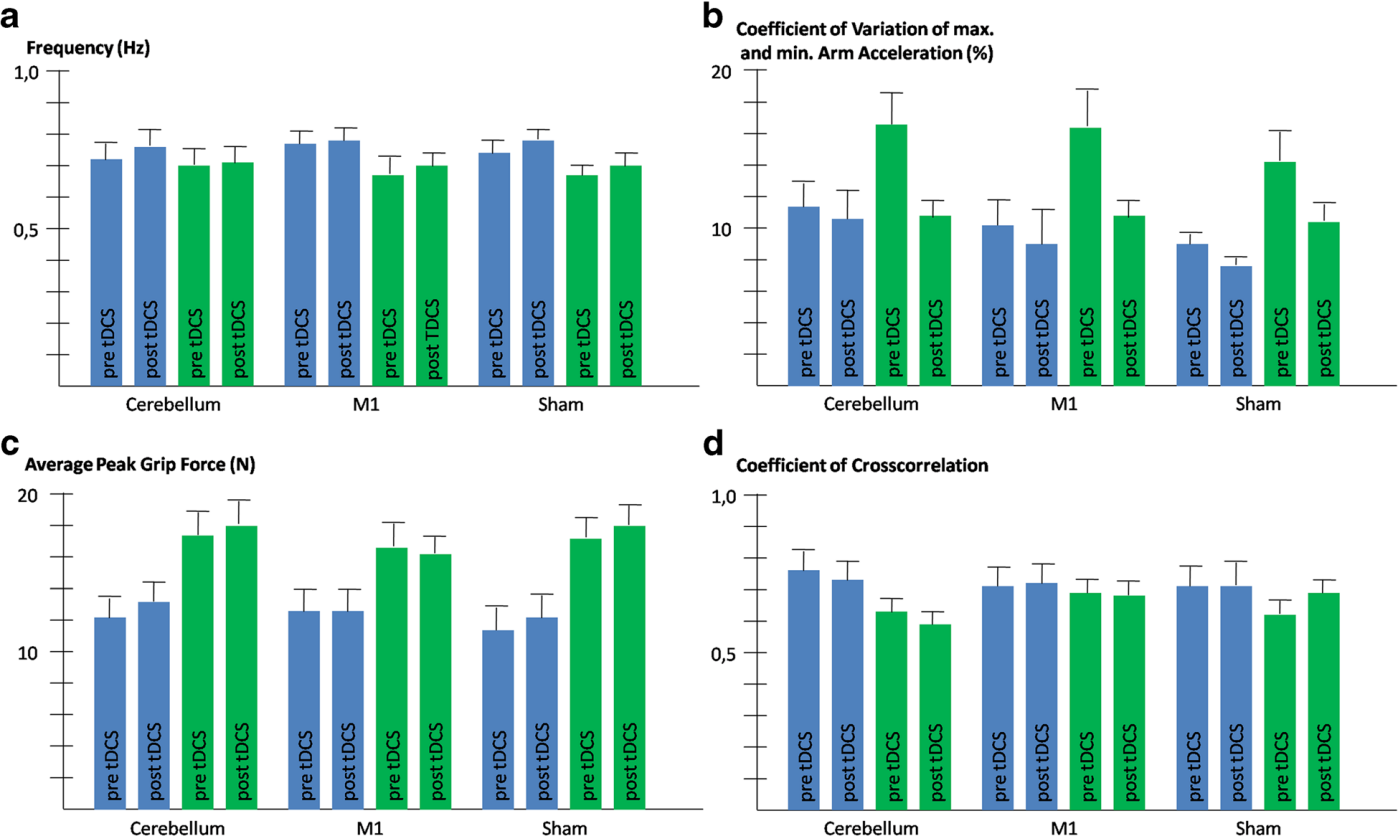
Effects of tDCS on Movement speed (**a**), variability of movements (**b**), grip force levels (**c**) and grip force-load force coupling (**d**). Blue bars = controls, green bars = cerebellar patients

Variability of arm movements was higher in patients through all conditions compared to controls (main effect of “group”: *F(1,24)* = 6.0, *P* = 0.022, see Fig. 2). Variability was lower post-stimulation for both groups (main effect of “time”: *F(1,24)* = 20.8, *P* < 0.001). Figure 2 and a statistically significant interaction between “time” and “group” (*F(1,24)* = 9.9, *P* = 0.004) indicates that the difference between groups was most prominent before the stimulations. Indeed the post-hoc test found a difference between patients and control subjects for the tests prior to tDCS (*t* = 2.6, *P* = 0.016), but not after tDCS (*P* > 0.1). No significant main effect nor any interaction were found for the factor “stimulation” (*P* > 0.1).

The average magnitude of peak grip forces exhibited by patients were clearly higher through all conditions compared to controls (main effect of “group”: *F(1,24)* = 11.0, *P* = 0.003, see Fig. 2). The analysis for the ratio of grip force to load force confirmed the findings for the absolute value for grip force (main effect of “group”: *F(1,24)* = 8.3, *P* = 0.008). No other factor nor any interaction reached statistical significance (*P* > 0.1).

The maximum coefficient of cross-correlation that measures the precision of the coupling between the grip force and the load force was higher in control subjects than in patients (see Fig. 2). The main effect of “group” was however not statistically significant (*P* > 0.1). The factor “group” was statistically significant for the interaction with “stimulation” (*F(2,50)* = 5.8, *P* = 0.005). Figure 2 suggests that particularly in the session with cerebellar stimulation, patients were less precise than control subjects. Pair-wise post-hoc tests detected a trend for this group difference (*t* = −2.0, *P* = 0.059), while no differences were obvious in the other stimulation conditions (*P* > 0.1). Furthermore, an interaction of “stimulation” x “time” was detected (*F(2,50)* = 3.3, *P* = 0.044). Figure 2 suggests that the coupling improved after the sham stimulation and deteriorated after cerebellar stimulation. However, pairwise post-hoc tests failed to prove difference between pre and post stimulation values in any of the three stimulation conditions (all *P* > 0.1). No interaction was found between “group” and “time” nor was the 3-way interaction between all factors significant. Thus, no differential effects of stimulation were evident in the patient group. There was no benefit on grip force control neither in cerebellar patients nor in controls following anodal tDCS applied over the cerebellum or M1.

## Discussion

Contrary to our hypothesis, there were no consistent tDCS effects on disordered grip-force control in cerebellar patients.

Patients with cerebellar degeneration exhibited higher grip-forces and higher variability of movements, which is in good accordance with previous studies [1, 7]. While impaired coupling of grip- and load-forces are also often observed in cerebellar patients [1, 6, 28] deficits in this measure were only present at a trend level in the current study. An increase in movement speed and acceleration post-stimulation was observed in patients and controls and irrespective of the stimulation condition and therefore likely attributed to practice effects. A further practice effects was evident for movement variability particularly in cerebellar patients. Possible reasons for the lack of cerebellar tDCS effects are discussed below.

Firstly, in the current study after-effects of tDCS were investigated. After-effects of tDCS have been detected up to 90 min following M1 stimulation based on changes in motor evoked potential amplitudes [29]. Most studies examining therapeutic effects of tDCS in cerebellar patients, including the studies conducted by Benussi et al. [11, 14], used off-line stimulation [30]. The influential studies by Galea et al. [10, 27], however, examined direct effects of tDCS, that is they applied an online stimulation approach. We cannot exclude that direct tDCS may have stronger effects on grip force control in cerebellar patients than tDCS after-effects.

Secondly, electrode positioning may have been suboptimal for the present task. Benussi et al. [11, 14], used a location which was centered in the midline, while we used a location centered over the right lateral cerebellum. A modelling study using the same electrode placement as we did, demonstrated current distribution mainly over the lateral posterior cerebellum sparing the vermis and intermediate cerebellum [31]. Yet, deficits in grip force control in degenerative cerebellar patients were associated with atrophy of the intermediate cerebellum [32]. The reach adaptation studies of Galea et al. [10, 27], on the other hand, showed clear effects of cerebellar tDCS using a similar electrode location as in the current study. An association of impaired prehensile movements has also been demonstrated with more lateral cerebellar areas in focal cerebellar patients [6, 28].

Thirdly, cerebellar tDCS effects may differ depending on the type of cerebellar degeneration. The pattern of cerebellar atrophy differs depending on the type of spinocerebellar ataxia, and extra-cerebellar areas are affected to various degrees [33, 34].

Fourthly, one must also consider variability of performance as a critical factor that could obscure intervention effects. While on average we succeeded to standardize the movement generated load profiles, individual trials deviated from the indented movement. Movements with relatively low frequency may have reduced the benefit of a precise coupling between grip force and load in control subjects and may therefore have been responsible for the missing group differences in coupling. Variability of the outcome measures may also have played a role. For example, it is difficult to reconcile the difference for coupling precision between patients and control subjects in the session with cerebellar stimulation compared to the other conditions. Since this interaction was independent of the time of testing, also the pre-stimulation data supported this effect. Therefore, variability of baseline performance in the cerebellar patients may have influenced this finding. Variability was however lower for the grip force and not even a tendency supporting any effect of stimulation was obvious. It therefore seems improbable that variability alone could explain the missing effects of stimulation.

Fifthly, we cannot exclude that the performance of the reach adaption task, which was performed during tDCS stimulation, had interfered with tDCS after-effects on grip force control.

Finally, cerebellar tDCS effects may be highly task dependent. Recently, Jalali et al. [35] did not replicate effects on cerebellar tDCS on visuomotor adaptation reported previously [10] when a range of task parameter were systematically varied. Besides, other studies report that cerebellar tDCS had no effects on motor learning in healthy controls and patients with cerebellar degeneration [26, 36]. Due to these inconsistencies it has been questioned whether cerebellar tDCS could become a valuable tool in clinical neurorehabilitation [26, 35].

Like cerebellar stimulation, M1-stimulation was not followed by significant effects on grip force control. There was a major difference in the setup used in a prior study reporting reduction of ataxia [17]. The authors used bilateral M1 stimulation. The anodal electrode was placed on the motor cortex contralateral to the most affected side and the cathode stimulation was placed on motor cortex of the less affected side of the body. In the present study the cathodal electrode was placed over the contralateral supraorbital region. It cannot be excluded that bilateral M1 stimulation leads to changes in grip force control. Reminiscent of the above findings, bilateral stimulation of the M1 lead to improved control of grip force during grasping and lifting of an object in stroke patients [22]. In a similar task tested in a sample of elderly subjects, unilateral stimulation of M1 also resulted in an increase efficiency of grip force control. However, results were not consistent. They were significant only for one of two object surface materials and not obvious for a temporal measure [21].

## Conclusion

No effects of cerebellar or M1 anodal tDCS were observed on grip force control in cerebellar patients. Further studies are needed to explore different stimulation parameters including online stimulation and /or optimized electrode placements. At present tDCS cannot be recommended in the neurorehabilitation of disordered grip force control in cerebellar disease.

## Abbreviations

AccZ: Vertical acceleration
ADCA: Autosomal dominant cerebellar ataxia
GF: Grip force
ICARS: International Cooperative Ataxia Rating Scale
LF: Load force
SAOA: Sporadic adult onset ataxia of unknown etiology
SARA: Scale for the Assessment and Rating of Ataxia
SCA: Spinocerebellar ataxia
SD: Standard deviation
tDCS: Transcranial direct current stimulation
TMS: Transcranial magnetic stimulation
